# Aversive learning reduces aversive-reinforcer sensitivity in honey bees

**DOI:** 10.1038/s41598-025-88267-y

**Published:** 2025-02-26

**Authors:** Yuan Lai, Stevanus Rio Tedjakumala, Luigi Baciadonna, Catherine Macri, Isabelle Lafon, Martin Giurfa

**Affiliations:** 1 Sorbonne Université, CNRS, Inserm, Neuro-SU, 75005 Paris, France; 2 Sorbonne Université, CNRS, Inserm, Institut de Biologie Paris-Seine, IBPS, 75005 Paris, France; 3 Research Centre on Animal Cognition, Centre of Integrative Biology, CNRS, University Paul Sabatier, 31062 Toulouse, France; 4 Institut Universitaire de France (IUF), Paris, France; 5 Present Address: Sartorius AG, 77694 Kehl, Baden-Württemberg Germany; 6 Present Address: CNRS, Délégation Occitanie Ouest, 31400 Toulouse, France

**Keywords:** Associative learning, Reinforcer processing, Aversive learning, Aversive-reinforcer sensitivity, Sting extension response, Honey bees, Animal behaviour, Learning and memory, Classical conditioning

## Abstract

Research on associative learning typically focuses on behavioral and neural changes in response to learned stimuli. In Pavlovian conditioning, changes in responsiveness to conditioned stimuli are crucial for demonstrating learning. A less explored, but equally important, question is whether learning can induce changes not only in the processing of conditioned stimuli but also in the processing of unconditioned stimuli. In this study, we addressed this question by combining reinforcer-sensitivity assays with Pavlovian conditioning in honey bees. We focused on aversive shock responsiveness, measuring the sting extension response to electric shocks of increasing voltage, and examined the effect of aversive olfactory conditioning—where bees learn to associate an odor with shock—on shock responsiveness. After experiencing electric shocks during conditioning, the bees showed a persistent decrease in responsiveness to lower voltages, observable three days after conditioning, indicating reduced shock sensitivity. This effect was specific to electric shock, as appetitive conditioning involving a sucrose reinforcer did not alter shock responsiveness, leaving shock sensitivity unchanged. These findings highlight a previously unexplored effect of associative learning on reinforcer sensitivity, demonstrating a lasting decrease of responsiveness to reinforcer intensities perceived as less relevant than that encountered during conditioning.

## Introduction

Associative learning enables individuals to identify predictive relationships in their environment, a critical ability for survival as it allows us to use past experiences to anticipate future events and adjust behavior accordingly^1^. Multiple short-, medium-, and long-term mechanisms drive the plastic changes in neural circuits underlying learning and memory formation^2,3^. Typically, analyses focus on behavioral and neural changes observed when learned stimuli are presented. In Pavlovian conditioning, for example, an initially neutral stimulus (the conditioned stimulus, or CS) gains the ability to elicit a conditioned response through its pairing with a biologically relevant stimulus (the unconditioned stimulus, or US)^4^. Since learning is defined by changes in CS processing, evidence of learning is usually based on altered responsiveness to the CS, as well as on the neural and molecular correlates of these changes^5^. However, assessing the response to the US both before and after conditioning is also essential in controlled experiments. High levels of US responsiveness before conditioning reflect proper motivation to engage in anticipatory learning of the US. Likewise, high levels of US responsiveness after conditioning indicate that the animal’s motivation remained intact throughout conditioning, ensuring that changes in CS responses can be attributed to learning itself, rather than to motivational shifts, fatigue, or sensory adaptation.

Studies on experience-dependent changes in unconditioned stimulus (US) processing are less common, as the subjective value of the US is generally assumed to remain constant during conditioning. For example, in Rescorla and Wagner’s model of Pavlovian learning^6^, the US establishes a fixed ceiling toward which conditioned responses converge over trials. In other words, perfect conditioning is achieved when the conditioned stimulus (CS) fully substitutes for the US. However, an animal’s experience with the US during learning trials may alter its perception of the US’s quality and intensity, though this possibility has been less explored. For instance, conditioning might create a specific expectation of a US with particular properties, leading to a change in the subjective evaluation of the US if its properties are modified before or after conditioning.

**Fig. 1:**
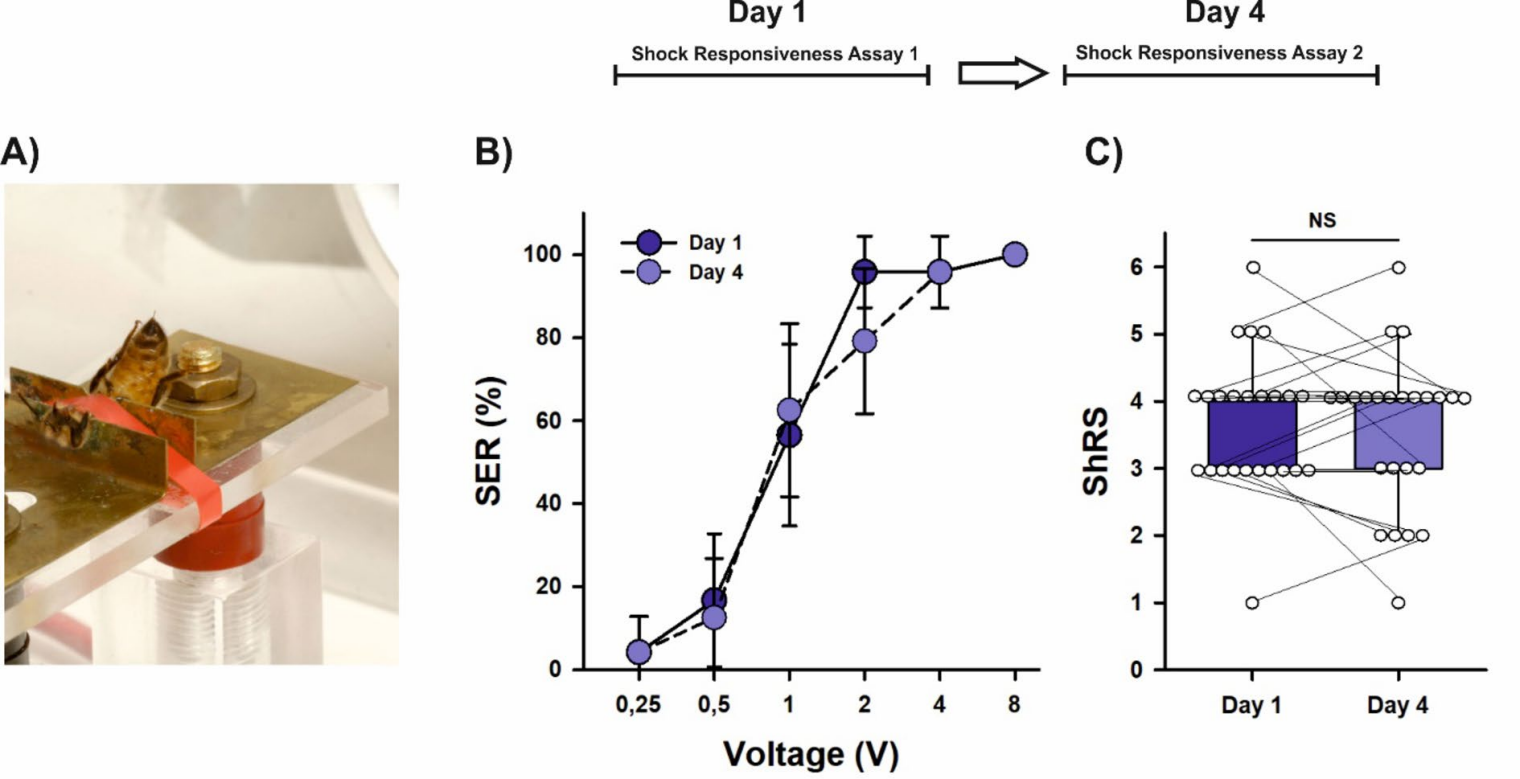
Aversive shock responsiveness remains unchanged from Day 1 to Day 4. (**A**) View of a honey bee in the setup used for aversive stimulation. The harnessed bee acts as a bridge between the two metallic plates fixed on a Plexiglas plate. EEG cream was smeared on the two notches of the metallic plates to ensure good contact between the plates and the bee. The bee closes a circuit and receives a mild electric shock which induces the sting extension reflex (SER). (**B**) Percentage of bees (*n* = 24) exhibiting sting extension response (SER) upon stimulation with a series of increasing voltages (0.25, 0.5, 1, 2, 4 and 8 V). Shock responsiveness was measured on Day 1 and then four days later on Day 4. No significant differences were detected between both responsiveness curves (GLMER, NS). Error bars indicate the 95% confidence interval. (**C**) Individual shock responsiveness scores (ShRS) did not vary from Day 1 to Day 4 (Wilcoxon test for paired samples, NS). For each bee a ShRS was calculated by determining the number of voltages to which it extended the sting upon stimulation with the complete series of voltages. ShRS could vary between 0 (no response) to 6 (response to all voltages). The graph shows the median and the 10th, 25th, 75th and 90th percentiles as vertical boxes with error bars. Dots indicate individual scores. Lines connect values for the same individuals. *NS* non-significant.

Honey bees offer a valuable opportunity to address this issue due to their suitability for studies on learning and memory^7–9^ as well as on US sensitivity^10,11^. In the laboratory, harnessed bees positioned between two small metal plates can be tested for aversive US sensitivity by measuring their responsiveness to a series of electric shocks of increasing voltages (Fig. 1A). The number of voltages that elicit the defensive, innate sting extension response (SER) serves as a measure of their aversive sensitivity^11,12^, with more sensitive bees responding to a greater range of voltages than less sensitive ones. Additionally, aversive Pavlovian olfactory conditioning can also be performed with honey bees^13–15^. In this protocol, harnessed bees are exposed to an odorant as the CS, which predicts an electric shock as the US; successful learners exhibit SER in response to the aversive olfactory CS and retain this memory for several days^13,15,16^.

It is therefore possible to assess a bee’s responsiveness to various intensities of an aversive reinforcer and determine whether subsequent aversive conditioning leads to persistent changes in its subjective evaluation of these reinforcer intensities. In this study, we conducted such an experiment by focusing on aversive shock responsiveness to see whether aversive olfactory conditioning, where bees learn to associate an odorant with a shock, induces long-term changes in shock responsiveness. The experiment consisted of three phases: (1) an initial phase evaluating aversive reinforcer sensitivity, in which responsiveness to electric shocks of increasing voltage was measured; (2) a phase of differential olfactory conditioning, where bees learned to discriminate between two odorants—one paired with a shock and the other unpaired; and (3) a final phase of re-evaluating aversive reinforcer sensitivity, performed three days after conditioning, where responsiveness to increasing voltages was reassessed. Additionally, we conducted a control experiment with a different group of bees, where the second phase involved appetitive differential olfactory conditioning^17,18^. In this case, one odorant was rewarded with a sucrose solution, while the other was not, allowing us to determine whether changes in shock responsiveness are induced by any form of conditioning or occur only when the same reinforcer type is consistent across phases. This approach aimed to identify whether repeated exposure to electric shock during conditioning induces lasting changes in how bees perceive different levels of this aversive reinforcer.

## Results

### Aversive shock responsiveness remains stable over time in non-conditioned bees

Since our main experimental design involves measuring aversive shock responsiveness on Day 1 and then again three days later, on Day 4, following aversive olfactory conditioning on Day 1, we first conducted a control experiment to determine whether shock responsiveness changes over time (from Day 1 to Day 4) in the absence of any conditioning.

To quantify shock responsiveness, we stimulated harnessed forager bees (*n* = 24) with a series of increasing voltages (0.25, 0.5, 1, 2, 4 and 8 V)^11,12^ and recorded the sting extension reflex (SER) after each stimulation (Fig. 1A). Shock responsiveness increased with higher voltages (Fig. 1B; percentage of bees responding to a given voltage; generalized linear mixed effects model [GLMER], χ^2^_(4)_ = 196.45, *p* < 2^e−16^) and remained consistent from Day 1 to Day 4, both at the population level (Fig. 1B; GLMER, χ^2^_(1)_ = 0.15, *p* = 0.42) and at the individual level (Fig. 1C; ShRS = Shock Responsiveness Scores, i.e., number of voltages that elicited a SER per individual; Wilcoxon test for paired samples; day 1 vs. day 4: z = − 0.659, *p* = 0.51). Therefore, shock responsiveness remains stable over time under our experimental conditions.

### Aversive learning induces a long-term decrease in shock sensitivity

**Fig. 2:**
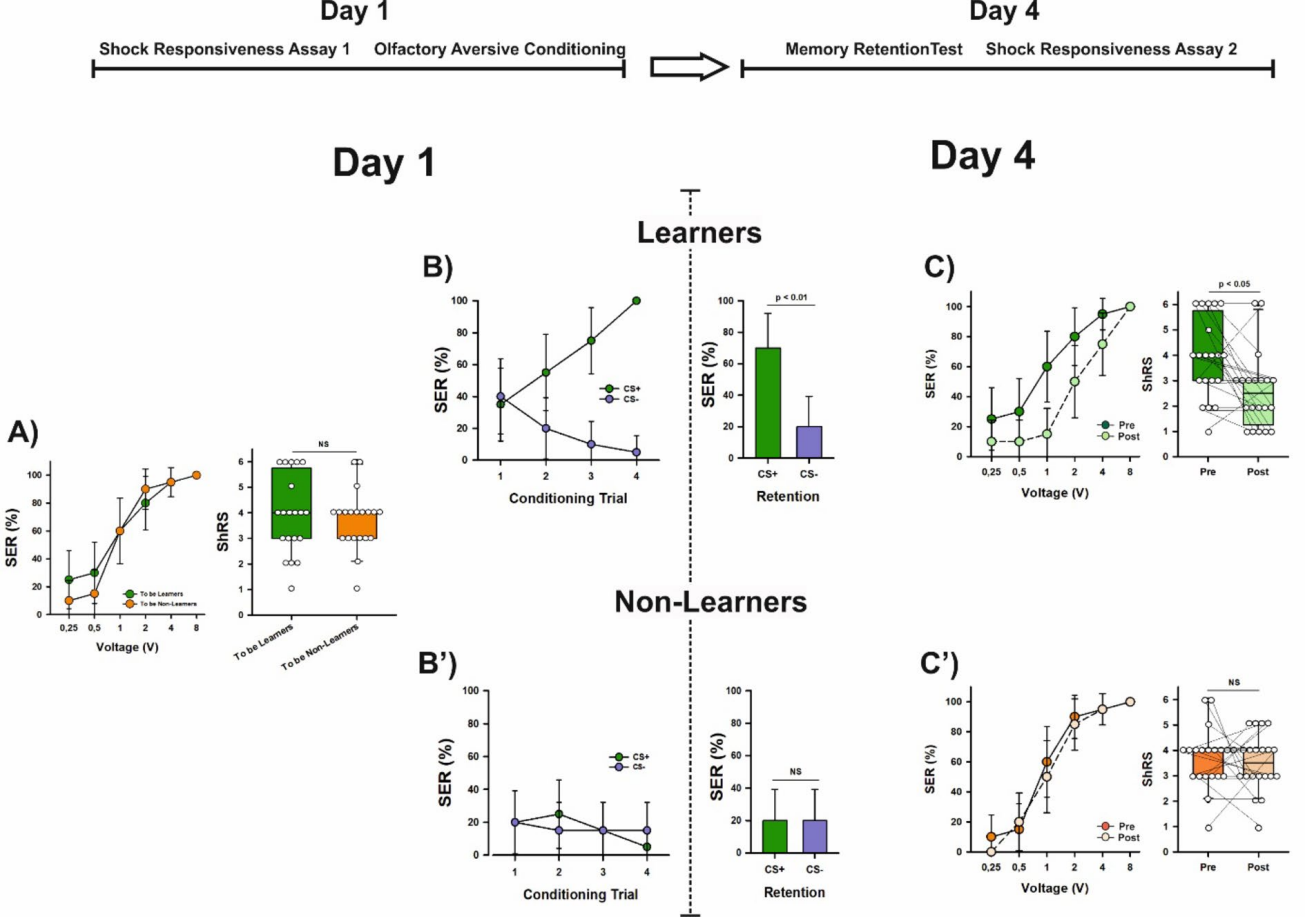
Aversive learning leads to a significant decrease in shock sensitivity. The upper part shows the experimental schedule. (**A**) Left: Percentage of bees (*n* = 40) exhibiting sting extension response (SER) upon stimulation with a series of increasing voltages (0.25, 0.5, 1, 2, 4 and 8 V) on Day 1. Shock responsiveness is shown for the two groups of bees which were later separated based on their learning performances (‘Learners’, *n* = 20, and ‘Non-Learners’, *n* = 20). No significant differences were detected between both groups (GLMER, NS). Error bars indicate the 95% confidence interval. Right: Individual shock responsiveness scores (ShRS) did not vary between both groups of bees (Mann–Whitney *U* test, NS). The graph shows the median and the 10th, 25th, 75th and 90th percentiles as vertical boxes with error bars. Dots indicate individual scores. *NS* non-significant. (**B**) Left: Acquisition curves of Learners (*n* = 20) in an aversive olfactory differential conditioning in which they had to differentiate between an odorant paired (CS+) with a 7.5 V electric shock and a non-punished odorant (CS−) along four CS+ and four CS− trials presented in pseudo-random order. Learners were defined as those bees showing more responses to the CS+ than to the CS− during the training and responding to the CS+ and not to the CS− in the last trial. The graph shows the % of bees responding with SER (conditioned responses) to the CS+ and to the CS−. Error bars indicate the 95% confidence interval. Right: Retention test performed on Day 4 (i.e. 3 days after conditioning). Bees were presented with the CS+ and the CS− in random order, which varied from bee to bee, and in the absence of shock. Bars indicate the % of bees responding with SER to the CS+ and to the CS−. Error bars indicate the 95% confidence interval. Learners mastered the olfactory discrimination on Day 1 and exhibited significant retention on Day 4. (**B**′) Left: Acquisition curves of Non-Learners (*n* = 20). Right: Retention test performed on Day 4 (i.e. 3 days after conditioning). Non- Learners did not master the olfactory discrimination on Day 1 and in consequence had no significant retention on Day 4. *NS* non-significant. (**C**) Left: Shock responsiveness of Learners on Day 4 (‘Post’ curve) compared to that on Day 1 (‘Pre’ curve; see **A**). Responsiveness decreased significantly after aversive learning (GLMER, *p* < 0.0001). Error bars indicate the 95% confidence interval. **Right**: Individual shock responsiveness scores (ShRS) decreased significantly between Day 1 (Pre; see **A**) and Day 4 (Post). The graph shows the median and the 10th, 25th, 75th and 90th percentiles as vertical boxes with error bars. Dots indicate individual scores. Lines connect values for the same individuals. (**C**′) Left: Shock responsiveness of Non-Learners on Day 4 (‘Post’ curve) compared to that on Day 1 (‘Pre’ curve; see **A**). Responsiveness did not change in the absence of learning (GLMER, NS). Error bars indicate the 95% confidence interval. Right: Individual shock responsiveness scores (ShRS) did not vary from Day 1 (Pre; see **A**) to Day 4 (Post). The graph shows the median and the 10th, 25th, 75th and 90th percentiles as vertical boxes with error bars. Dots indicate individual scores. Lines connect values for the same individuals. *NS* non-significant.

We measured shock responsiveness in a different group of forager bees (*n* = 40) using the same procedure described previously. As in the earlier experiment, shock responsiveness increased with increasing voltages (Fig. 2A; GLMER, χ^2^_(4)_ = 183.69, *p* < 2^e−16^) and reached 100% at 8 V. At this point, the two groups of bees—later classified based on their learning performance—showed no difference in sting responsiveness at the population level (Fig. 2A; GLMER, χ^2^_(1)_ = 0.24, *p* = 0.95) or in their individual ShRS (Fig. 2A; Mann–Whitney *U* test, z = 0.33, *p* = 0.75).

Four hours after measuring shock responsiveness, the bees underwent differential SER conditioning, where they were trained to discriminate 1-nonanol (CS+), which was paired with a 7.5 V electric shock, from 1-hexanal, which was not paired with a shock (CS−). Each bee served as its own control, learning to respond to the CS+ and not to the CS−. Four CS+ and four CS− trials were presented in a pseudo-random order. Bees that responded more to the CS+ than to the CS− during training and responded to the CS+ but not to the CS− in the final trial were classified as ‘learners’ (*n* = 20). Those that did not meet these criteria were classified as ‘non-learners’ (n = 20)^19^.

Learners successfully discriminated the CS+ from the CS− (Fig. 2B; factor CS; Wilcoxon for paired samples, V = 0, *p* = 1.85^e−4^) across trials (Fig. 2B; factor trial; Friedman test, χ^2^_(7)_ = 60, *p* = 1.53^e−10^), while non-learners failed to make this discrimination (Fig. 2B′; factor CS; Wilcoxon for paired samples, V = 52, *p* = 1) across trials (Fig. 2B′; factor trial; Friedman test, χ^2^_(7)_ = 3.5, *p* = 0.83), despite undergoing the same conditioning protocol.

Memory retention was assessed three days after conditioning (Day 4), a post-training interval associated with long-term memory dependent on protein synthesis in aversive olfactory conditioning^15^. To test memory retention, bees were presented with the CS+ and the CS− in a random sequence that varied for each bee, without any shocks. Memory retention was significant in learners, which responded more to the CS+ than to the CS− in the absence of reinforcement (Fig. 2B; McNemar’s test, χ^2^ = 8.10, *p* < 0.01). As expected, non-learners did not show a difference in their responses to the CS+ and CS− (Fig. 2B′; χ^2^ = 0.5, *p* = 0.5, NS).

Shock responsiveness was measured immediately after the retention test for both learners and non-learners. Learners showed an overall increase in shock responsiveness with increasing voltages (Fig. 2C; Friedman test, χ^2^_(4)_ = 55.4, *p* = 2.67^e−11^). However, their responsiveness was lower than before conditioning. This decrease was evident not only at the population level (Fig. 2C; responsiveness pre vs. post learning: Wilcoxon test for paired samples, V = 18, *p* = 0.017) but also in individual responsiveness scores (Fig. 2C; Wilcoxon test for paired samples; ShRS pre vs. post learning: z = 2.414, *p* = 0.016), indicating that after aversive conditioning, individual bees responded to fewer voltage levels (median ShRS pre conditioning: 4; median ShRS score post conditioning: 2.5).

To explore this change in responsiveness at the population level, we compared pre- and post-learning responsiveness for each voltage (Bonferroni correction 0.05/5 = 0.01). This analysis revealed that the percentage of learners responding to intermediate voltages was significantly lower after conditioning, particularly at 1 V (pre vs. post learning % SER for 1 V: Wilcoxon for paired samples, V = 6, *p* = 0.007; all the other comparisons ≥ 0.04).

In non-learners, an expected increase in responsiveness with increasing voltages was also observed (Fig. 2C′; Friedman test, χ^2^_(4)_ = 67.2, *p* = 8.86^e−14^). However, their responsiveness did not differ from pre-conditioning levels, either at the population level (Fig. 2C′; pre vs. post learning: Wilcoxon test for paired samples, V = 47.5, *p* = 0.77), or in individual responsiveness scores (Fig. 2C′; z = 0.54, *p* = 0.59) despite being subjected to the same conditioning protocol and sensory stimulations. This result shows that the acquisition of odor-shock associations significantly reduced shock responsiveness, particularly at intermediate voltages lower than those used during conditioning.

**Fig. 3:**
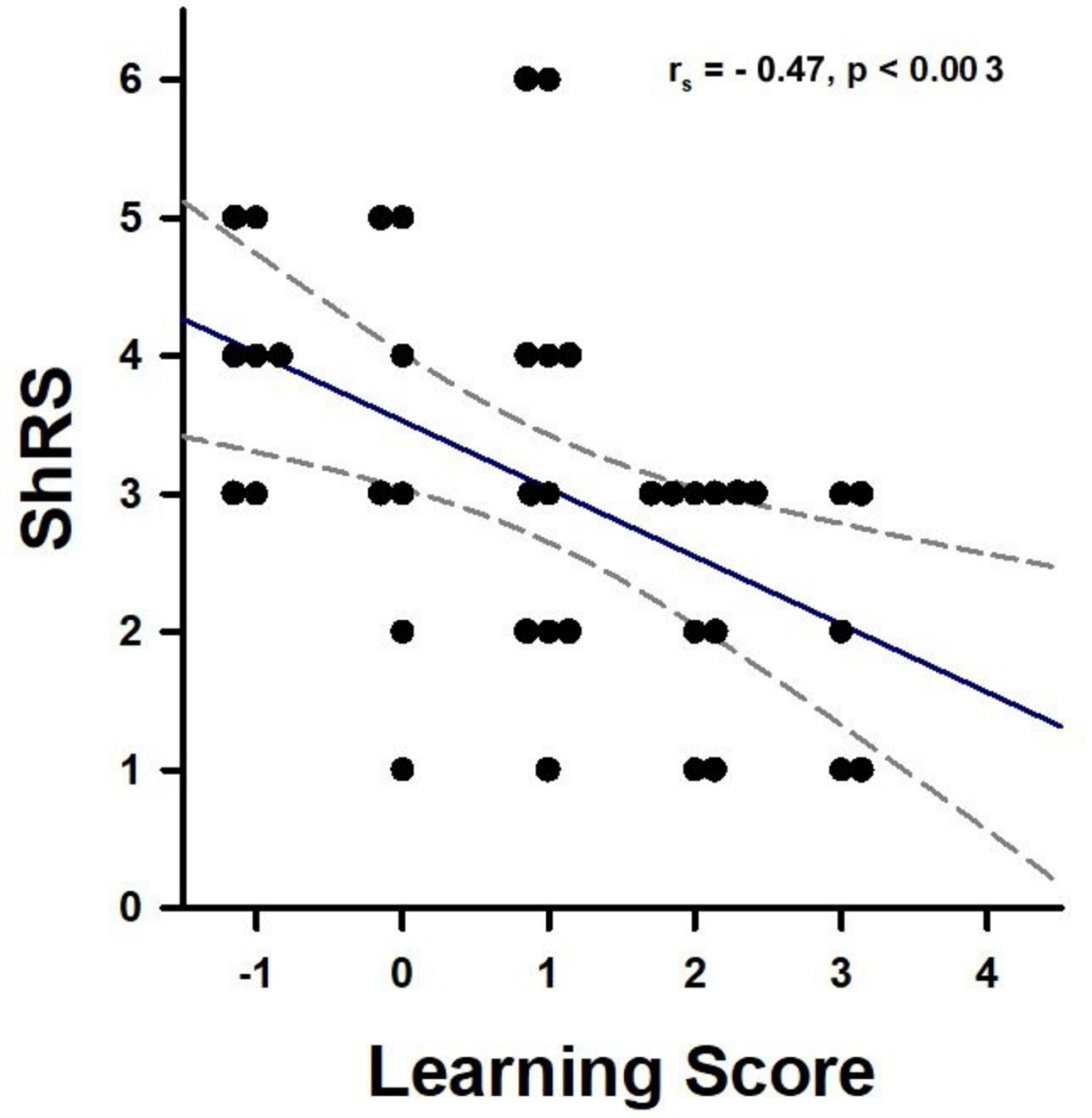
Correlation between individual aversive learning scores and individual shock responsiveness scores. Data for both Learners (*n* = 20) and Non-Learners (*n* = 20) are shown. The two variables were inversely correlated (Spearman’s Correlation analysis, *p* < 0.003).

To confirm this conclusion, we calculated a learning score for both groups of bees (‘learners’ and ‘non-learners’). The score ranged from 3 to − 3, with a higher positive score indicating successful learning. The score was based on the last three CS+/CS− trials, as random responses are expected during the first CS+/CS− trial. We then analyzed whether learning scores were inversely correlated with subsequent shock responsiveness scores (ShRS) on Day 4, which would indicate a decrease in aversive responsiveness following aversive conditioning. Indeed, both scores were inversely correlated at the individual level (Fig. 3; Spearman’s Correlation analysis; r_s_ = − 0.47; p = 0.003), confirming that better aversive learning resulted in a greater decrease in shock responsiveness.

### Aversive learning, not memory retrieval, drives the long-term decrease in shock sensitivity

In the previous experiment, a memory retrieval test was conducted on Day 4 prior to reassessing shock responsiveness for the second time. One could, therefore, argue that the observed decrease in shock responsiveness on Day 4 was due to the retrieval of an aversive memory, which might have influenced the animals’ internal state and, consequently, their sensitivity to shock. To test this hypothesis, we repeated the previous experiment but omitted the memory retrieval test on Day 4.

**Fig. 4:**
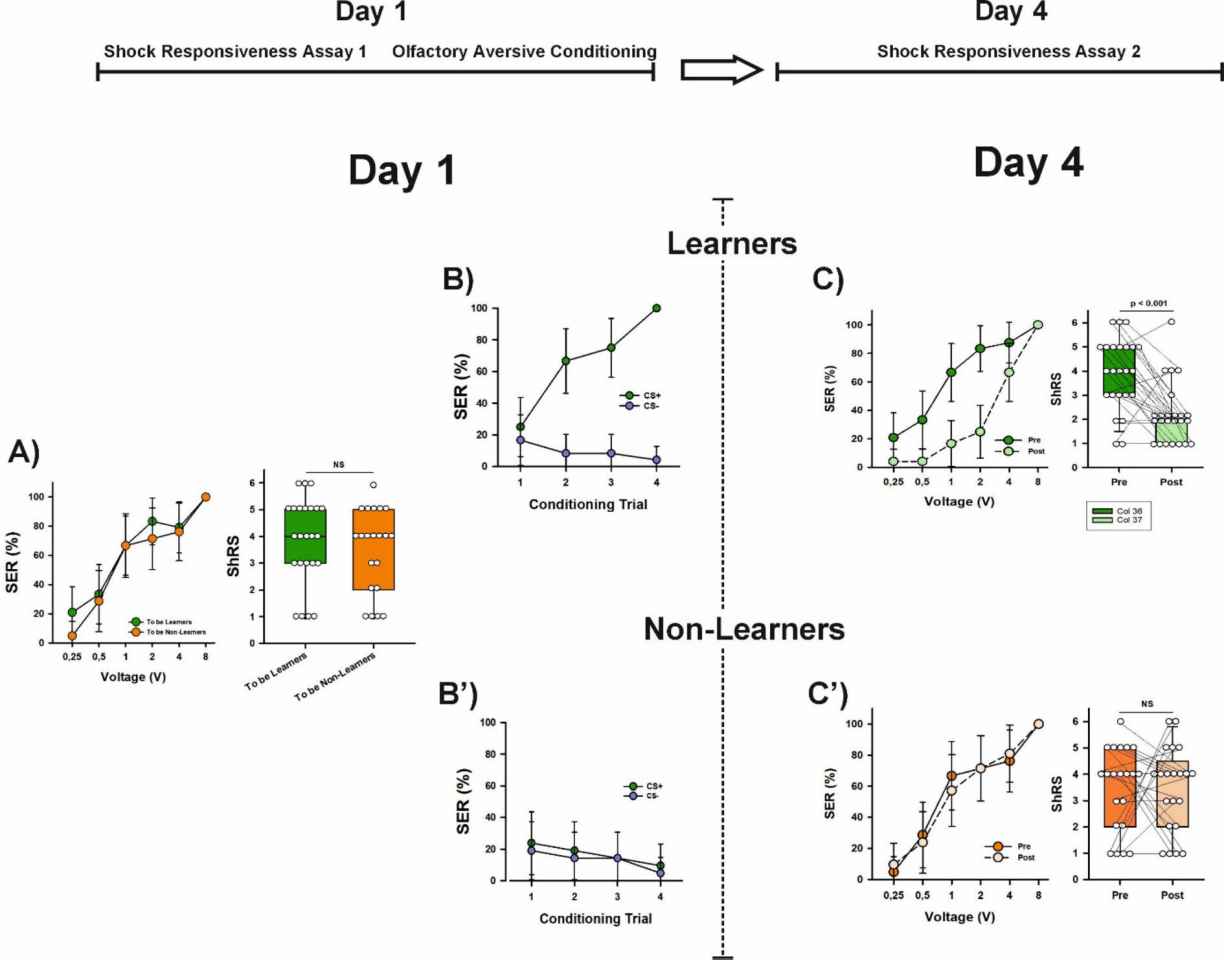
Aversive Learning, not memory retrieval, drives the long-term decrease in shock sensitivity. The upper part shows the experimental schedule, which omitted the memory-retrieval test on Day 4. (**A**) Left: Percentage of bees (*n* = 45) exhibiting sting extension response (SER) upon stimulation with a series of increasing voltages (0.25, 0.5, 1, 2, 4 and 8 V) on Day 1. Shock responsiveness is shown for the two groups of bees which were later separated based on their learning performances (‘Learners’, *n* = 24, and ‘Non-Learners’, *n* = 21). No significant differences were detected between both groups (GLMER, NS). Error bars indicate the 95% confidence interval. Right: Individual shock responsiveness scores (ShRS) did not vary between both groups of bees (Mann–Whitney *U* test, NS). The graph shows the median and the 10th, 25th, 75th and 90th percentiles as vertical boxes with error bars. Dots indicate individual scores. *NS* non-significant. (**B**) Acquisition curves of Learners (*n* = 24) in an aversive olfactory differential conditioning in which they had to differentiate between an odorant paired (CS+) with a 7.5 V electric shock and a non-punished odorant (CS−) along four CS+ and four CS− trials presented in pseudo-random order. Learners were defined as those bees showing more responses to the CS+ than to the CS− during the training and responding to the CS+ and not to the CS− in the last trial. The graph shows the % of bees responding with SER (conditioned responses) to the CS+ and to the CS−. Error bars indicate the 95% confidence interval. Learners mastered the olfactory discrimination on Day 1. (**B**′) Acquisition curves of Non-Learners (*n* = 21). Non- Learners did not master the olfactory discrimination on Day 1. (**C**) Left: Shock responsiveness of Learners on Day 4 (‘Post’ curve) compared to that on Day 1 (‘Pre’ curve; see **A**). Responsiveness decreased significantly after aversive learning (*p* < 0.0001). Error bars indicate the 95% confidence interval. Right: Individual shock responsiveness scores (ShRS) decreased significantly between Day 1 (Pre; see **A**) and Day 4 (Post). The graph shows the median and the 10th, 25th, 75th and 90th percentiles as vertical boxes with error bars. Dots indicate individual scores. Lines connect values for the same individuals. (**C**′) Left: Shock responsiveness of Non-Learners on Day 4 (‘Post’ curve) compared to that on Day 1 (‘Pre’ curve; see **A**). Responsiveness did not change in the absence of learning (NS). Error bars indicate the 95% confidence interval. Right: Individual shock responsiveness scores (ShRS) did not vary from Day 1 (Pre; see **A**) to Day 4 (Post). The graph shows the median and the 10th, 25th, 75th and 90th percentiles as vertical boxes with error bars. Dots indicate individual scores. Lines connect values for the same individuals. *NS* non-significant.

Shock responsiveness was measured in a new group of bees (*n* = 45). As in the previous experiment, responsiveness increased with increasing voltages (Fig. 4A; GLMER χ^2^_(4)_ = 112.63, *p* < 2^e−16^) and reached 100% at 8 V. The two groups of bees—later differentiated based on their learning performance—showed no difference in shock responsiveness at the population level (Fig. 4A; GLMER, χ^2^_(1)_ = 1.07, *p* = 0.30) or in their individual ShRS (Fig. 4A; Mann–Whitney *U* test, z = − 0.73, *p* = 0.47).

Four hours after measuring shock responsiveness, the bees underwent differential SER conditioning, where they were trained to discriminate 1-nonanol (CS+) paired with a 7.5 V electric shock from 1-hexanal, which was not paired with a shock (CS−). We used the same criteria as in the previous experiment to distinguish between ‘learners’ (*n* = 24) and ‘non-learners’ (n = 21).

Learners successfully discriminated the CS+ from the CS− (Fig. 4B; factor CS; Wilcoxon test for paired samples, V = 0, *p* = 1.85^e−4^) across trials (Fig. 4B; factor trial; Friedman test, χ^2^_(7)_ = 60, *p* = 1.53^e−10^), while non-learners failed to make this discrimination (Fig. 4B′; factor CS; Wilcoxon test for paired samples, V = 43, *p* = 0.54) across trials (Fig. 4B′; factor trial; Friedman test, χ^2^_(7)_ = 4.09, *p* = 0.77).

Shock responsiveness was assessed three days after conditioning (Day 4), this time without conducting a retention test beforehand. Learners showed an overall increase in shock responsiveness with increasing voltages (Fig. 4C; Friedman test, χ^2^_(4)_ = 63.5, *p* = 5.38^e−13^). However, their responsiveness was lower than before conditioning. This decrease was observed not only at the population level (Fig. 4C; responsiveness pre vs. post learning: Wilcoxon test for paired samples, V = 16, *p* = 5.59^e−4^) but also in individual responsiveness scores (Fig. 4C; Wilcoxon test for paired samples; ShRS pre vs. post learning: z = 3.319, *p* = 0.0009), confirming that after aversive conditioning decreased individual shock responsiveness (median ShRS pre conditioning: 4; median ShRS score post conditioning: 2).

To explore this change in responsiveness at the population level, we compared pre- and post-learning responsiveness for each voltage (Bonferroni correction 0.05/5 = 0.01). This analysis revealed that the percentage of learners responding to intermediate voltages was significantly lower after conditioning, particularly at 1 and 2 V (pre vs. post learning % SER for *1 V*: Wilcoxon test for paired samples, V = 17, *p* = 0.002; pre vs. post learning % SER for *2 V*: Wilcoxon test for paired samples, V = 19, *p* = 0.001; all the other comparisons ≥ 0.02).

In non-learners, an expected increase in responsiveness with increasing voltages was also observed (Fig. 4C′; Friedman test, χ^2^_(4)_ = 59.9, *p* = 3^e−12^). However, their responsiveness did not differ from pre-conditioning levels, either at the population level (Fig. 4C′; pre vs. post learning: Wilcoxon test for paired samples, V = 81.5, *p* = 0.87), or in individual responsiveness scores (Fig. 4C′; z = 0.196, *p* = 0.84) despite being subjected to the same conditioning protocol and sensory stimulations. This result shows that the acquisition of odor-shock associations, and not the retrieval of the memory of these associations, significantly reduced shock responsiveness, particularly at intermediate voltages lower than those used during conditioning.

**Fig. 5:**
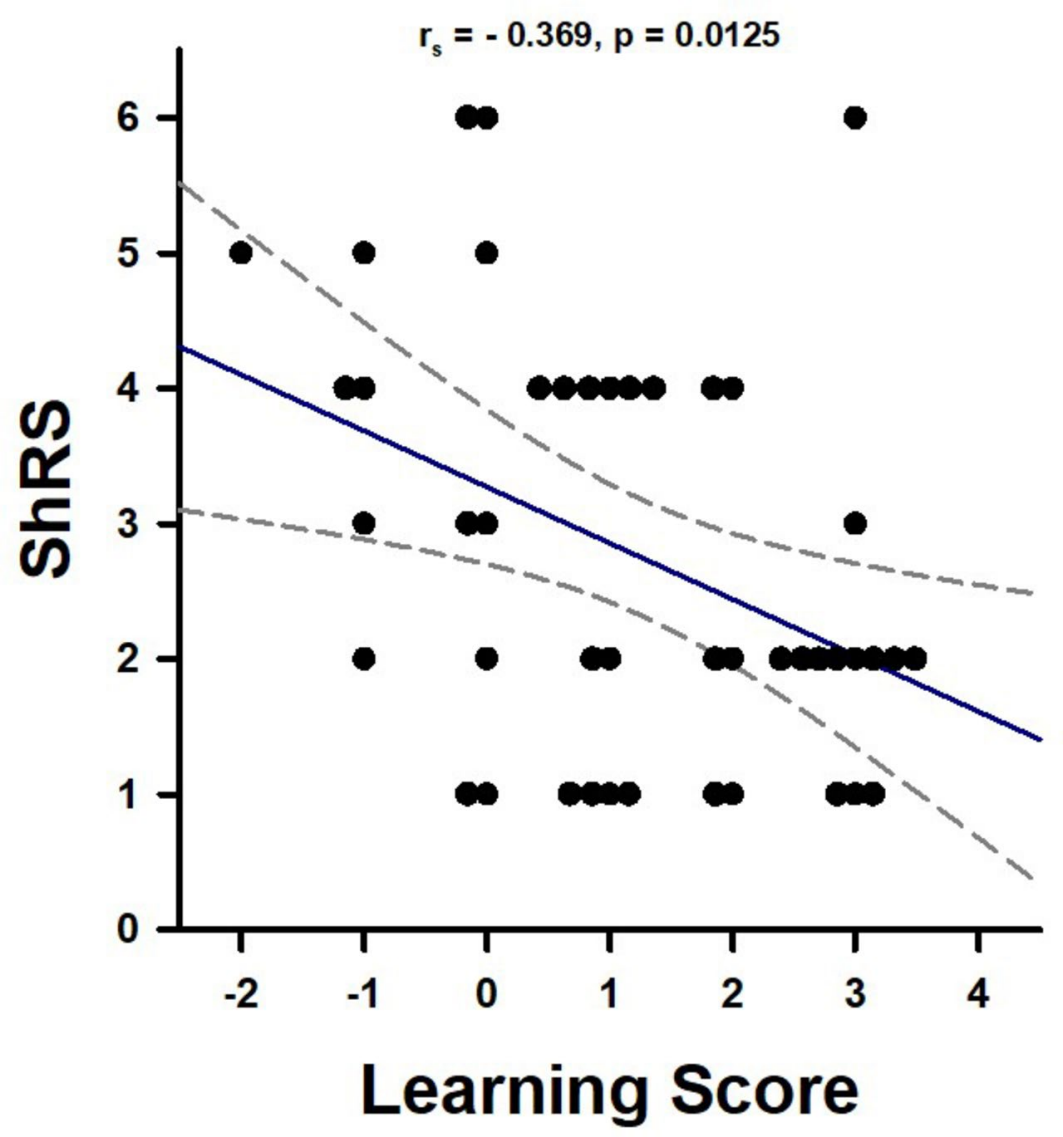
Correlation between individual aversive learning scores and individual shock responsiveness scores in the absence of a memory-retrieval test. Data for both Learners (*n* = 24) and Non-Learners (*n* = 21) are shown. The two variables were inversely correlated (Spearman’s Correlation analysis, *p* = 0.0012).

Finally, we analyzed whether learning scores were inversely correlated with Day-4 shock responsiveness scores (ShRS), confirming a decrease in aversive responsiveness as a consequence of aversive conditioning. As in the previous experiment, both scores were inversely correlated (Fig. 5; Spearman’s Correlation analysis; r_s_ = − 0.369; *p* = 0.0125), confirming that better performances in aversive learning resulted in a greater decrease in shock responsiveness.

### Appetitive learning does not induce a decrease in aversive shock sensitivity

We then asked whether the long-term modification of aversive responsiveness induced by aversive conditioning is specific to that form of conditioning, given the common use of electric shock in all experimental phases, or if it is also induced by a different form of conditioning interspersed between the two phases of aversive-reinforcer sensitivity evaluation.

To answer this question, we investigated the effect of appetitive olfactory conditioning on aversive responsiveness in a different group of bees. On Day 1, harnessed bees were exposed to the same series of increasing voltages as in the previous experiment (0.25, 0.5, 1, 2, 4, and 8 V), followed by appetitive differential PER conditioning. In this phase, bees were trained to discriminate 1-nonanol (CS+), which was paired with a 40% (w/w) sucrose solution, from 1-hexanal, which was not paired with the sucrose solution. Three days after conditioning (Day 4), the bees were tested for memory retention by presenting the CS+ and CS− in a random order for each bee. Finally, the bees were retested for aversive responsiveness immediately after the retention test (Day 4) by exposing them again to the same series of increasing voltages as on Day 1.

**Fig. 6:**
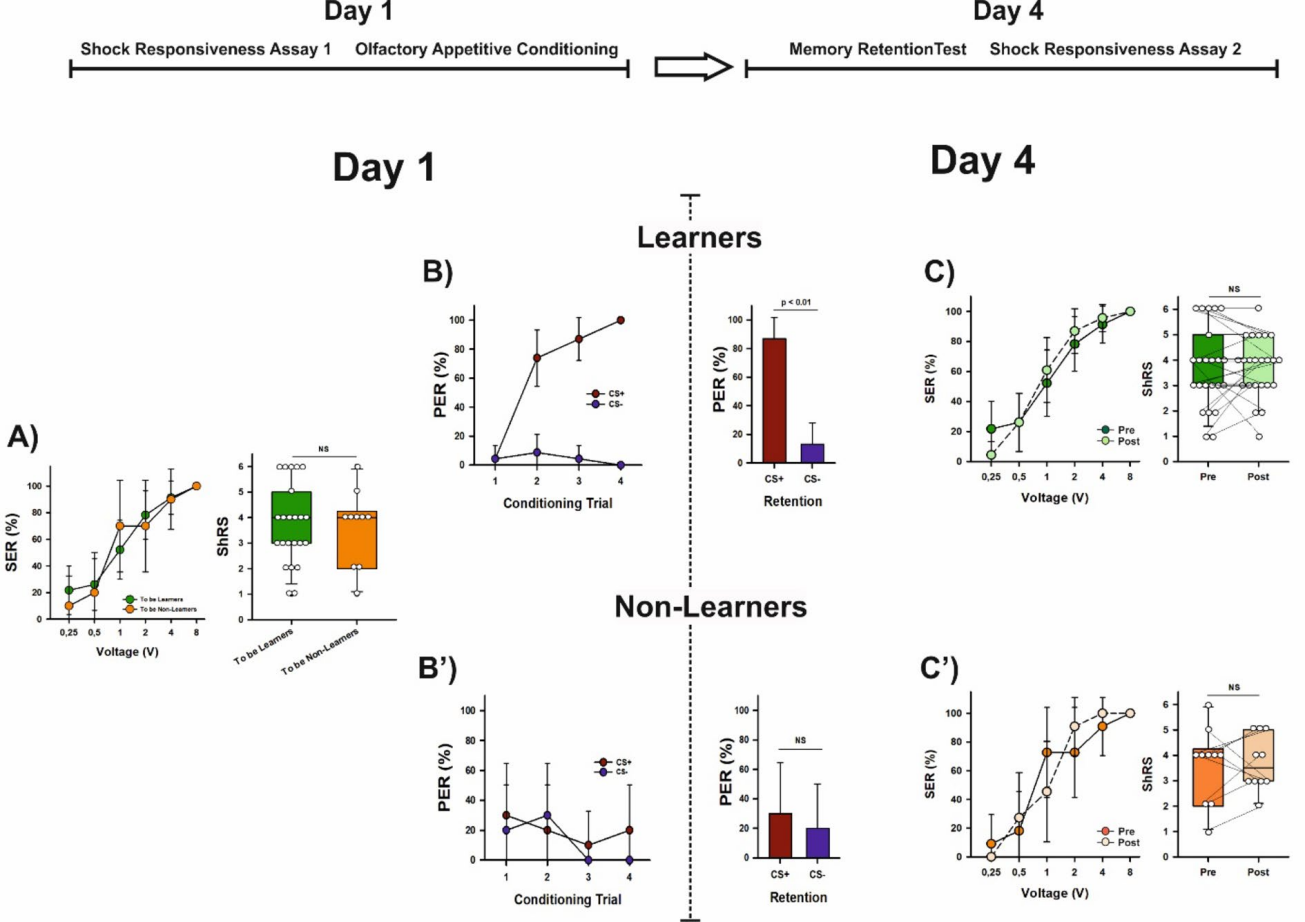
Appetitive learning does not alter shock sensitivity. The upper part shows the experimental schedule. (**A**) Left: Percentage of bees (*n* = 33) exhibiting sting extension response (SER) upon stimulation with a series of increasing voltages (0.25, 0.5, 1, 2, 4 and 8 V) on Day 1. Shock responsiveness is shown for the two groups of bees which were later separated based on their learning performances (‘Learners’, *n* = 23, and ‘Non-Learners’, *n* = 10). No significant differences were detected between both groups (GLMER, NS). Error bars indicate the 95% confidence interval. Right: Individual shock responsiveness scores (ShRS) did not vary between both groups of bees (Mann–Whitney *U* test, NS). The graph shows the median and the 10th, 25th, 75th and 90th percentiles as vertical boxes with error bars. Dots indicate individual scores. *NS* non-significant. (**B**) Left: Acquisition curves of Learners (*n* = 23) in an appetitive olfactory differential conditioning in which they had to differentiate between an odorant rewarded with sucrose solution (CS+) and a non-rewarded odorant (CS−) along four CS+ and four CS− trials presented in pseudo-random order. Learners were defined as those bees showing more responses to the CS+ than to the CS− during the training and responding to the CS+ and not to the CS− in the last trial. The graph shows the % of bees responding with PER (conditioned responses) to the CS+ and to the CS−. Error bars indicate the 95% confidence interval. Right: Retention test performed on Day 4 (i.e. 3 days after conditioning). Bees were presented with the CS+ and the CS− in random order, which varied from bee to bee, and in the absence of sucrose solution. Bars indicate the % of bees responding with PER to the CS+ and to the CS−. Error bars indicate the 95% confidence interval. Learners mastered the olfactory discrimination on Day 1 and exhibited significant retention on Day 4. (**B**′) Left: Acquisition curves of Non-Learners (*n* = 10). Right: Retention test performed on Day 4 (i.e. 3 days after conditioning). Non- Learners did not master the olfactory discrimination on Day 1 and in consequence had no significant retention on Day 4. *NS* non-significant. (**C**) Left: Shock responsiveness of Learners on Day 4 (‘Post’ curve) compared to that on Day 1 (‘Pre’ curve; see **A**). Responsiveness did not change after appetitive learning (GLMER, NS). Error bars indicate the 95% confidence interval. Right: Individual shock responsiveness scores (ShRS) did not vary significantly between Day 1 (Pre; see **A**) and Day 4 (Post). The graph shows the median and the 10th, 25th, 75th and 90th percentiles as vertical boxes with error bars. Dots indicate individual scores. Lines connect values for the same individuals. (**C**′) Left: Shock responsiveness of Non-Learners on Day 4 (‘Post’ curve) compared to that on Day 1 (‘Pre’ curve; see **A**). Responsiveness did not change in the absence of learning (GLMER, NS). Error bars indicate the 95% confidence interval. Right: Individual shock responsiveness scores (ShRS) did not vary from Day 1 (Pre; see **A**) to Day 4 (Post). The graph shows the median and the 10th, 25th, 75th and 90th percentiles as vertical boxes with error bars. Dots indicate individual scores. Lines connect values for the same individuals. *NS* non-significant.

On Day 1, shock responsiveness increased with increasing voltages and reached 100% at 8 V, as in previous experiments (Fig. 6A; GLMER, χ^2^_(4)_ = 139.80, p = < 2^e−16^). At this point, the two groups of bees, which were later separated based on their different learning performances, did not differ in sting responsiveness at the population level (Fig. 6A; GLMER, χ^2^_(1)_ = 0.04, *p* = 0.86) or in their individual ShRS (Fig. 6A; Mann–Whitney *U* test, z = − 0.196, *p* = 0.98).

Four hours after quantifying shock responsiveness, the bees underwent appetitive differential olfactory conditioning. They were trained to discriminate 1-nonanol (CS+), paired with a 40% (w/w) sucrose solution, from 1-hexanal, which was not paired with sucrose. The bees remained harnessed in the same holders used for shock delivery, allowing odorants to be delivered to their antennae and the sucrose solution to both their antennae and proboscis. Learners and non-learners were defined using the same criteria as for the aversive differential conditioning (see above). Learners (*n* = 23) successfully discriminated between the two odorants, extending their proboscis to the CS+ and not to the CS− (Fig. 6B; factor CS; Wilcoxon test for paired samples, V = 0, *p* = 2.07^e−5^) across trials (factor trial; Friedman test, χ^2^_(7)_ = 119, *p* = 1.05^e−22^), while non-learners (*n* = 10) failed to achieve olfactory discrimination (Fig. 6B′; factor CS; Wilcoxon test for paired samples, V = 4, *p* = 0.40) across trials (factor trial; Friedman test, χ^2^_(7)_ = 6.83, *p* = 0.44).

On Day 4, appetitive memory retention was assessed by presenting both groups of bees with the CS+ and the CS− in a random order, without sucrose solution. Memory retention was significant in learners (Fig. 6B; χ^2^ = 15.06, *p* < 0.0001), while non-learners showed no difference in their PER to the CS+ and the CS−, as expected given the absence of observable learning (Fig. 6B′; χ^2^ = 0.0, *p* = 1.0, NS).

After assessing memory retention, the bees were re-tested for shock responsiveness as on Day 1. As expected, learners showed increased responsiveness with increasing voltages (Fig. 6C; Friedman test, χ^2^_(4)_ = 66.2, *p* = 1.45^e−13^). However, their responses did not differ from those observed before appetitive conditioning, either at the population level (Fig. 6C; responsiveness pre vs. post learning: Wilcoxon test for paired samples, V = 69.5, *p* = 0.60), or in their individual responsiveness scores (Fig. 6C; Wilcoxon test for paired samples; ShRS pre vs. post learning: z = 0.283, *p* = 0.78). Similarly, non-learners also showed increased shock responsiveness with increasing voltages (Fig. 6C′; Friedman test, χ^2^_(4)_ = 33.2, *p* = 1.09^e−6^) and their responsiveness remained unchanged, both at the population level (Fig. 4C′: pre vs. post learning: Wilcoxon test for paired samples, V = 33, *p* = 0.69) and at the individual level (Fig. 6C′: ShRS pre vs. post learning: z = 0.152, *p* = 0.88) after conditioning. Therefore, appetitive associative learning, where sucrose solution was used as a reinforcer, did not alter sensitivity to electric shocks. The decrease in sensitivity to electric shocks with voltages lower than that used for conditioning (Figs. 2C and 4C) is reinforcer-specific and is not observed when sucrose solution is the reinforcer used during conditioning.

**Fig. 7:**
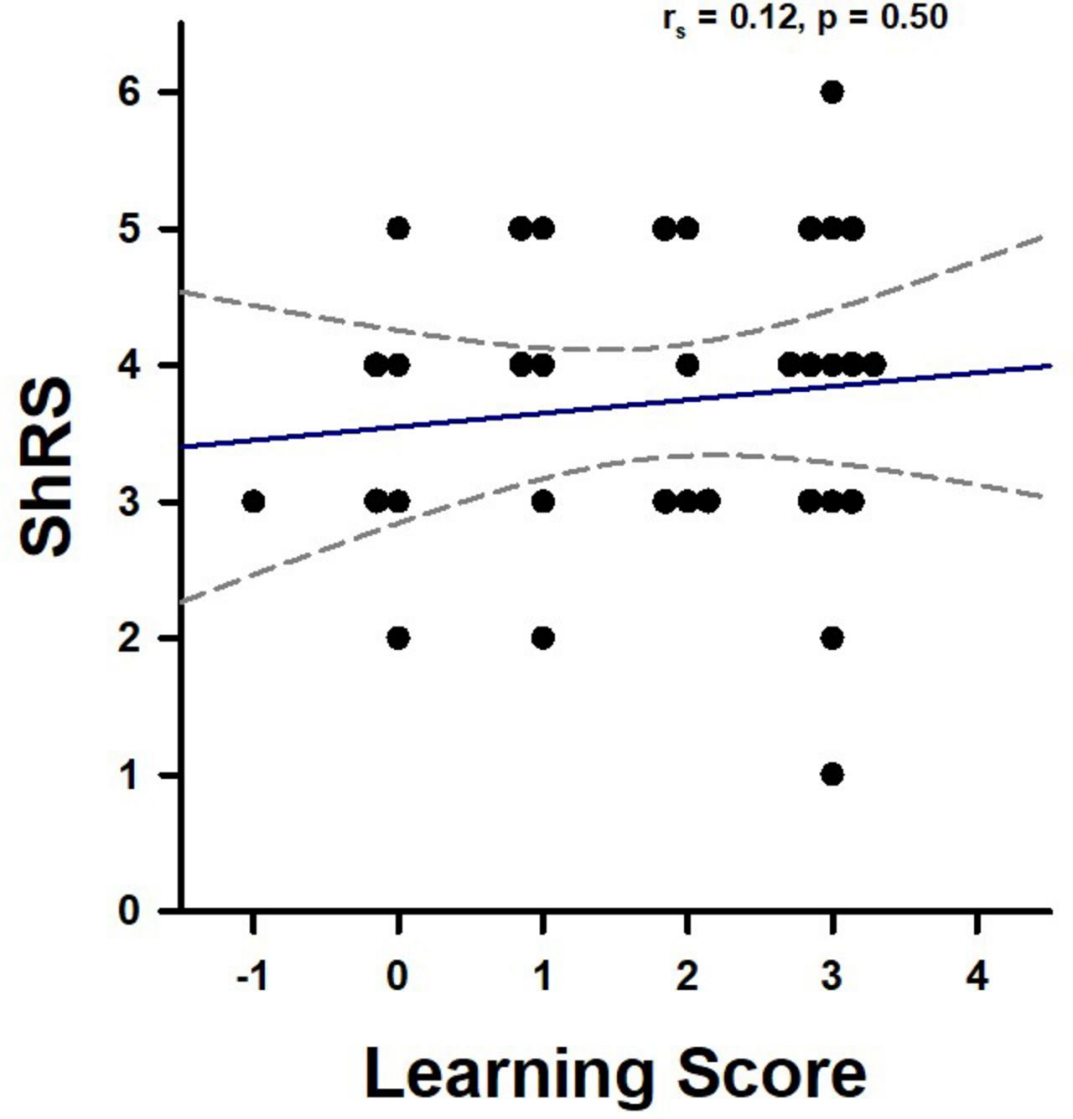
Correlation between individual appetitive learning scores and individual shock responsiveness scores. Data for both Learners (*n* = 23) and Non-Learners (*n* = 10) are shown. The two variables were not significantly correlated (Spearman’s Correlation analysis, *p* = 0.50).

We validated this conclusion through a correlation analysis between the appetitive olfactory learning scores and the subsequent shock responsiveness scores (ShRS) recorded on Day 4. Unlike the results observed in aversive olfactory conditioning (Figs. 3 and 5), no significant correlation was detected between the two individual scores (Fig. 7; Spearman’s correlation analysis: (r_s_ = 0.12; *p* = 0.5026), confirming the lack of influence of appetitive learning on aversive shock responsiveness.

## Discussion

Our results show that sensitivity to aversive stimuli changes as a consequence of aversive conditioning, in which the same type of reinforcer is administered. After experiencing predictable, repeated electric shocks at a voltage of 7.5 V, which is close to the maximum voltage used to assess shock sensitivity (8 V), bees showed a persistent decreased overall responsiveness to lower voltages. This effect was particularly evident at intermediate voltages (e.g., 1 V and 2 V), while the lowest voltages (0.2 V and 0.5 V) initially elicited minimal responses. This effect was specific to electric shocks, as interspersing a phase of appetitive conditioning between the pre- and post-evaluations of shock responsiveness did not alter the bees’ response to the shocks.

### A long-lasting change in the subjective evaluation of reinforcement as a result of learning

These results suggest that during conditioning, bees not only learned to associate the conditioned stimulus (CS) with an electric shock, but they also developed long-term, specific expectations about the aversive reinforcement delivered. The reduction in shock responsiveness was induced by the acquisition process (Fig. 4), and not by the memory retention test, and persisted three days after conditioning, a period during which olfactory memories formed through aversive olfactory conditioning are stabilized via transcription and translation processes.

No change in their responsiveness to the unconditioned stimulus (US) itself was observed. However, when bees experienced shocks with lower voltages after conditioning, they showed reduced responsiveness compared to the responsiveness observed in naïve bees three days after conditioning. This suggests that when bees form an associative memory between the CS and US and learn the predictive link between both stimuli, they also memorize the intensity of the US. This memory of the US intensity is responsible for reducing responses to lower-intensity US.

While the decrease in voltage responsiveness was evident across all four lowest voltages (0.25, 0.5, 1, and 2 V; see Fig. 2C, left panel), the statistical method employed detected a significant difference only at 1 V in Fig. 2C and at 1 V and 2 V in Fig. 4C. This can be explained by the fact that responses to the lowest voltages (0.25 and 0.5 V) were initially very low—reflecting the minimal stimulation provided at these levels. As a result, the subsequent decrease induced by aversive learning likely could not go any lower due to a bottom threshold effect. Nonetheless, the analysis of shock responsiveness scores in the learners (Figs. 2C and 4C, right panel) confirmed a significant reduction in responsiveness after aversive conditioning, aligning with the lower responsiveness levels observed in the responsiveness curve of the learners.

This result indicates that a long-lasting change in the subjective evaluation of reinforcement occurred as a result of learning: the bees became less sensitive to reinforcement intensities lower than those used during conditioning. These lower voltages were likely perceived as more tolerable compared to the higher voltage experienced during conditioning. This explanation aligns with the fact that a change in shock responsiveness was not observed after appetitive olfactory conditioning, which used sucrose solution as the US. The long-lasting change in shock responsiveness -observable three days after conditioning- appears specific to the use of electric shock during conditioning.

Delivering a voltage higher than that used during conditioning may have the opposite effect—an increase in responsiveness—since it would be perceived as less tolerable compared to the voltage used in conditioning. Although this reasoning is sound, such an experiment presents the challenge of using a voltage so high that it could harm the bees’ physical integrity. In fact, the use of 7.5 V for conditioning was determined through experiments that optimized a balance between inducing consistent learning and repeated sting extension response (SER) while preserving the physical integrity of the shocked individuals^14^. Lowering the conditioning voltage (e.g., to 4 V) in order to evaluate the response to 8 V is also impractical because conditioning is significantly less effective at lower voltages^14^, making it difficult to assess the effect of learning on responsiveness to 8 V.

The generalizability of the present findings could be tested by examining the impact of olfactory appetitive PER (proboscis extension reflex) conditioning^17,20^ on appetitive responsiveness, which is typically measured by PER to increasing concentrations of sucrose solution^10,21–24^. Our results suggest that after appetitive olfactory conditioning, which generally uses a 50% (w/w) sucrose solution as the unconditioned stimulus (US), responsiveness to concentrations lower than 50% may decrease. This experiment would also address an additional question: is associative learning necessary to induce a decrease in sucrose responsiveness? Unlike aversive olfactory SER (sting extension reflex) conditioning, where learning is most effective under a differential conditioning regime, absolute appetitive PER conditioning (i.e., conditioning with a single odorant) produces excellent learning performance. A characteristic ‘limitation’ of this conditioning form (for a comparison between learners and non-learners) is the consistently low percentage of non-learners (as seen, for example, in the experiment using appetitive conditioning in the present study; Fig. 6). Yet, the use of absolute conditioning allows for the inclusion of US-only control groups, which can help determine the influence of non-associative factors. The experiment would reveal whether non-associative exposure to an appetitive US also reduces responsiveness to lower concentrations of sucrose.

Another question is whether the changes in aversive responsiveness caused by aversive conditioning are specific to the aversive reinforcer used during conditioning or whether they extend to other aversive reinforcers. In other words, does the decrease in voltage responsiveness persist when a different aversive reinforcer is used during conditioning? This could be tested using aversive thermal stimulation, which also elicits SER in harnessed bees^25^. Bees can learn to associate an odorant with this aversive stimulus and respond with SER to the odorant that predicts the thermal US^26^. This experiment could help determine whether this type of associative learning also reduces shock responsiveness or whether the decrease is specific to electric shocks.

### Neural mechanisms underlying changes in aversive reinforcer sensitivity due to aversive learning

The neural mechanisms underlying this change in responsiveness remain to be determined. However, some specific hypotheses can be proposed based on previous findings about the neural basis of shock responsiveness and aversive olfactory conditioning. Both behavioral contexts share a common feature: the role of dopaminergic signalling. This signalling mediates the reinforcing properties of electric shock during olfactory conditioning^14^, and acts as a depressor of sting responsiveness to electric shocks so that when its effect is antagonized, responsiveness increases^12^. It has been suggested that different classes of dopaminergic neurons are responsible for these two functions—one functioning as an instructive system in olfactory conditioning, providing valence information to odorants in an associative, aversive learning context^27^, and another downregulating responsiveness, thereby acting as a gain control system^12^. Dopaminergic neurons are abundant in the bee brain, and some of them exhibit the necessary connectivity to perform these functions^28^, including specific connections within the olfactory circuit and broad branching across the brain to modulate various motivational components and sensory modalities.

Among the three types of dopaminergic receptors identified in honey bees (AmDOP1, AmDOP2 and AmDOP3), AmDOP3 appears to be a promising candidate to explain the decreases in shock responsiveness observed in our study. While AmDOP1^29,30^ is related to vertebrate D1-like receptors and increases cAMP levels when stimulated by dopamine, AmDOP2^30,31^, although also increasing cAMP upon dopaminergic stimulation, is more related to octopamine receptors and differs from vertebrate D1-like receptors. On the other hand, AmDOP3^32^ is related to vertebrate D2-like receptors and thus decreases intracellular cAMP levels upon dopamine stimulation. This reduction in cAMP via AmDOP3-signaling could be linked to the decrease in shock responsiveness observed after aversive olfactory conditioning. In this context, aversive conditioning may lead to an increase in AmDOP3 signalling, for instance, through upregulation of AmDOP3 receptors. This hypothesis could be tested by comparing levels of *AmDop1* to *AmDop3* receptor gene expression in the brains of learners and non-learners at the end of the experimental sequence used in this study.

### Ecological consequences of changes in reinforcer sensitivity due to aversive learning

Finally, the ecological implications and context of these findings warrant consideration. The observed reduction in responsiveness to less intense noxious stimuli following aversive learning may play a critical role in prioritizing defensive behaviors toward more significant threats. This mechanism could be vital for individual survival, particularly since stinging can be fatal for bees if their sting becomes lodged in the elastic tissue of larger predators, such as mammals^33^. By diminishing responses to less dangerous stimuli, bees may conserve their defensive resources for truly serious threats. This selective response helps avoid expending energy and risking injury on stimuli initially perceived as threats but later deemed irrelevant through learning. More broadly, the associative learning-driven decrease in reinforcer sensitivity may enhance selectivity for higher-intensity reinforcers, thereby optimizing both individual and colony performance.

Various environmental and physiological/genetic factors have been identified as modulators of innate responsiveness in bees, particularly in relation to appetitive responsiveness (PER to sucrose solution). Sucrose responsiveness is influenced by factors such as age, sex, and caste^22^, genotype^22,34^, season^35^, nutrition, foraging experience^21^ and exposure to pheromones^24,36^, among others. In the case of shock responsiveness, factors such as caste and age^33^, stress due to a shortage of colony resources^37^, the absence of a queen^38,39^, the presence of predators^40^ and the perception of alarm pheromones triggering attacks influence defensive responsiveness^41,42^. To this list, we now add associative learning, which, in the case of shock responsiveness, adjusts sensitivity to respond to highly relevant stimuli.

## Methods

### Insects

Honey bee (*Apis mellifera*) foragers were captured at an artificial feeder to which they were previously trained. These bees, typically older than 5–7 days, have a fully developed sting reflex^43^ and show increased responsiveness to electric shocks^11^. The feeder was located 20 m from the hive and contained a 40% (w/w) sucrose solution. Honey bee queen mates with several drones in a nuptial flight, thus resulting in various patrilines co-existing within a colony^44^. Thus, the variability existing in our data is intrinsic to natural colonies.

After capture, bees were brought to the laboratory and chilled on ice for 4 min until immobilized. They were then marked with number tags for identification and placed in individual harnesses (Fig. 1) designed for aversive stimulation *via* electric shock^14,15,45^. The harnesses consisted of two brass plates attached to a Plexiglas base, with the brass plates connected to a stimulator output (60 Hz AC current). The resistance measured between the plates when a bee was present ranged from 200 to 300 kΩ. Conductive gel was applied below the thorax to ensure effective shock delivery. Once secured, each bee was fed with a droplet (5 µl) of sucrose solution 30% (w/w) and allowed to rest for 1.5 h in an incubator set to 32 °C with 60–70% humidity.

### Shock responsiveness assay

Shock responsiveness was measured twice (on Day 1 and Day 4) by recording the occurrence of the sting extension response (SER). Harnessed bees were subjected to electric shocks of increasing voltages applied in ascending order: 0.25, 0.5, 1, 2, 4, and 8 V. Each shock trial lasted 20 s, consisting of 10 s of familiarization in the setup, followed by a 2-s electric shock, and then 8 s of post-shock rest before the bee was replaced by the next test subject. The interval between trials was 10 min. Full sting extension was scored as 1, while no response or partial responses were scored as 0^11^. For each voltage, we recorded the percentage of bees exhibiting SER in response to the electric shock.

The shock responsiveness score (ShRS) for each bee was calculated as the sum of all responses across the entire voltage range. This score could vary from 0 (no response) to 6. For example, a bee that extended its sting in response to voltages from 1 to 8 V would have an aversive score of 4, having responded to four consecutive voltages. Bees that exhibited inconsistent responses—i.e., responding at a lower voltage but not at higher ones—were excluded from the analysis, as their aversive scores would be unreliable^11^.

### Aversive olfactory conditioning

On Day 1, after measuring shock responsiveness, the bees underwent aversive differential olfactory conditioning, where they were trained to discriminate between an odor associated with a shock (CS+) and an odor not paired with a shock (CS−). The aversive unconditioned stimulus (US) was a 7.5 V electric shock applied for 2 s. The CS+ was 1-nonanol, and the CS− was 1-hexanal (both from Sigma Aldrich, Deisenhofen, Germany). Five microliters of pure odorants were applied to 1 cm^2^ pieces of filter paper, which were then placed inside 20 ml syringes for odor delivery to the bees’ antennae. Each odorant was presented for 5 s. An air extractor behind the bee prevented odor accumulation and minimized possible contamination from pheromone release.

Each conditioning trial lasted 1 min. The bee was placed at the stimulation site in front of the air extractor and left there for 20 s before being exposed to the odorant paired with the electric shock. The shock began 3 s after the odorant onset and ended with the odorant. Afterward, the bee remained in the setup for 35 s before being removed. The intertrial interval (ITI) was always 10 min. Each bee served as its own control, learning to respond to the CS+ and not to the CS−. A total of four CS+ and four CS− trials were presented in pseudo-random order. Bees that showed more responses to the CS+ than to the CS− during the conditioning trials, and responded to the CS+ but not to the CS− in the final trial, were classified as ‘learners’. Those that did not meet these criteria were considered ‘non-learners’^19^. For each trial, we recorded the percentage of bees responding with the sting extension response (SER) to both the CS+ and CS−. Bees that did not respond to the US during conditioning trials were excluded from the experiments. Thus, all bees (both learners and non-learners) exhibited the sting extension response to the US in every conditioning trial.

For each bee, a learning score was calculated based on performance during the last three trials, as a random response is expected in the first CS+/CS− trial. A correct response to the CS+ was given a score of +1, while no response was scored as 0. For CS− trials, a lack of response was scored as 0, while an incorrect response was assigned a score of − 1. Learning scores could thus range from 3 to − 3, with higher positive scores indicating more successful learning.

Retention tests were conducted on Day 4, three days after the conditioning. During these tests, the CS+ and CS− were presented in a random sequence (which varied for each bee), without the electric shock. The ITI between odor presentations was 10 min. In all experiments, responses to the 7.5 V shock were measured before and after conditioning or retention tests. Only bees that consistently responded to the electric shock were included in the analyses.

### Appetitive olfactory conditioning

On Day 1, after measuring shock responsiveness, bees maintained in the holders used for shock delivery underwent appetitive differential olfactory conditioning. In this conditioning, they learned to discriminate between an odor associated with a sucrose reward (CS+) and a non-rewarded odor (CS−). The appetitive unconditioned stimulus (US) was a 40% (w/w) sucrose solution, which was first applied to the antennae to elicit the proboscis extension response (PER), and then to the proboscis itself for 2 s. The CS+ was 1-nonanol, and the CS− was 1-hexanal (Sigma Aldrich, Deisenhofen, Germany). Five microliters of the pure odorants were applied to 1 cm^2^ pieces of filter paper, which were then placed in 20 ml syringes to deliver the odor to the antennae^17^. Each odor was presented for 5 s. An air extractor positioned behind the bee prevented the accumulation of odorants. Each conditioning trial lasted 1 min. The bee was placed at the stimulation site, in front of the air extractor, for 20 s before being exposed to the odor paired with the sucrose solution. US delivery began 3 s after the odor onset and ended with the odor presentation. The bee remained in the setup for an additional 35 s before being removed. The intertrial interval (ITI) was always 10 min. Each bee acted as its own control, as it had to learn to respond with PER to the CS+ and not to the CS−. Four CS+ and four CS− trials were presented in a pseudo-random order. Bees that showed more responses to the CS+ than to the CS− during the conditioning trials, and responded to the CS+ but not to the CS− in the last trial, were classified as “learners.” Bees that did not meet these criteria were classified as “non-learners”. For each conditioning trial, we recorded the percentage of bees responding with PER to both the CS+ and the CS−. Bees that did not respond to the US during conditioning trials were excluded from the experiments. Thus, all bees (both learners and non-learners) exhibited the proboscis extension response to the sucrose solution used as US in every conditioning trial.

For each conditioned bee, a learning score was calculated based on the last three trials, as random responses were expected during the first CS+/CS− trial. The score was determined by assigning a value of +1 for a correct response to the CS+ and 0 for no response. During CS− trials, a score of 0 was assigned for no response and −1 for an incorrect response. Learning scores ranged from 3 to − 3, with higher positive scores indicating more successful learning.

Retention tests were conducted on Day 4, three days after conditioning. The tests involved presenting the CS+ and CS− in a random sequence, which varied from bee to bee, without the sucrose solution. The ITI between odor presentations was 10 min. In all experiments, US responses to the 40% sucrose solution were measured both before and after the conditioning or retention tests. Only bees that consistently responded to the sucrose solution were included in the analysis.

### Experimental sequence

All experiments lasted four days. On Day 1, individually marked bees were harnessed as described, fed with 5 µl of 30% sucrose solution, and kept to rest for 1.5 h in an incubator (as mentioned above). Afterward, they underwent the first shock responsiveness assay.

In the control experiment, where shock responsiveness was measured again on Day 4 without any additional manipulation in between, the individually marked bees were chilled on ice for approximately 3 min after the first responsiveness assay to release them from the holders and clean the gel on their backs. The bees were then placed in small cages that had been cleaned with 70% ethanol and rinsed with distilled water. Groups of 10 to 20 bees were housed in these cages, which had two vertically positioned Eppendorf tubes filled with 30% sucrose solution. A small hole at the tip of each tube allowed the bees to drink the solution. The tubes were replaced with fresh ones daily, and any dead bees were removed at that time. The cages were stored in an incubator at 32 °C and 60–70% humidity in a room illuminated with red light. On the morning of Day 4, the cages were chilled for about 3 min, and the bees were re-harnessed in their individual holders. They were fed 5 µl of 30% sucrose solution and rested for 1.5 h before undergoing the second shock responsiveness assay.

In experiments where a conditioning assay followed the initial shock responsiveness assay on Day 1, the interval between the two assays was only 4 h. Due to this relatively short time gap, the individually marked bees remained in their holders between assays. Once conditioning was completed, the bees were chilled on ice for approximately 3 min to release them from the holders and clean the gel on their backs. They were then placed in small cages and treated as described above. On the morning of Day 4, the cages were chilled for about 3 min so the bees could be re-harnessed in their individual holders. They were fed 5 µl of 30% sucrose solution and rested for 1.5 h before the retention test. The second shock responsiveness assay was conducted an average of 10–15 min after the retention test.

### Quantification and statistical analysis

We performed three independent generalized linear mixed models (GLMER) using the lme4 package in R 4.3.2 software^46^. In the first GLMER we included SER (binomial distribution) as response variable and day (two levels: day1 and day4), voltage (four levels: 0.25, 0.5, 1, 2 and 4 V) and their interaction as fixed factors. In the second and third GLMER we included SER (binomial distribution) as response variable and category (two levels: being a learner and not being a learner), tension (four levels: 0.25, 0.5, 1, 2 and 4) and their interaction as fixed factors. In all cases, the fifth level of voltage (8 V) was not included as it was always 100% for the groups to be compared. Bee identity was included as a random factor to control for repeated measurements of the same subject in all models run. Each model was compared using the MuMin package^47^, and the model with the lower Akaike information criterion (AIC) was selected. The p-value of each factor was derived using the “drop1” function^48^. Based on the model selection criteria, the interaction effect was removed for all GLMER analyses.

Wilcoxon test for paired data was used to compare the SER or PER response to CS+ versus CS− in aversive or appetitive olfactory differential conditioning. Friedman’s test was used instead to compare SER or PER responses across differential conditioning trials or across the different voltage series used. Memory retention performances were analyzed by means of a McNemar’s test. Individual shock responsiveness scores (ShRS) of independent groups were compared using a Mann–Whitney *U* test. Responsiveness scores before and after conditioning of the same group of bees were compared by means of a Wilcoxon test for paired samples.

A Spearman’s correlation coefficient was calculated using the lm function in R 4.3.2 software to assess the relationship between Learning Score and post-conditioning ShRS for all the bees (Learners and NonLearners).

Statistical analyses were conducted using the R 4.3.2 software (R project) and Statistica 13.3 (Tibco Software).

## Data Availability

The raw data that support the findings in this paper are openly available at: 10.6084/m9.figshare.26889607.
